# Epigallocatechin-3-Gallate Ameliorated Iron Accumulation and Apoptosis and Promoted Neuronal Regeneration and Memory/Cognitive Functions in the Hippocampus Induced by Exposure to a Chronic High-Altitude Hypoxia Environment

**DOI:** 10.1007/s11064-022-03611-2

**Published:** 2022-05-13

**Authors:** Chen Chen, Bo Li, Haotian Chen, Yuhui Qin, Junying Cheng, Bo He, Yixuan Wan, Dongyong Zhu, Fabao Gao

**Affiliations:** 1 Department of Radiology, West China Hospital, Sichuan University, 37 Guoxue Road, Chengdu, 610041 Sichuan People’s Republic of China; 2 Department of MRI, the First Affiliated Hospital of Zhengzhou University, Zhengzhou, People’s Republic of China

**Keywords:** High altitude, Hypoxia, Quantitative susceptibility mapping, Iron accumulation, Drug intervention

## Abstract

We aimed to explore the protective effects and potential treatment mechanism of Epigallocatechin-3-gallate (EGCG) in an animal model of chronic exposure in a natural high-altitude hypoxia (HAH) environment. Behavioral alterations were assessed with the Morris water maze test. Iron accumulation in the hippocampus was detected by using DAB enhanced Perls’ staining, MRI, qPCR and colorimetry, respectively. Oxidative stress (malondialdehyde, MDA), apoptosis (Caspase-3), and neural regeneration (brain-derived neurotrophic factor, BDNF) were detected by using ELISA and western blotting. Neural ultrastructural changes were evaluated by transmission electron microscopy (TEM). The results showed that learning and memory performance of rats decreased when exposure to HAH environment. It was followed by iron accumulation, dysfunctional iron metabolism, reduced BDNF and the upregulation of MDA and Caspase-3. TEM confirmed the ultrastructural changes in neurons and mitochondria. EGCG reduced HAH-induced cognitive impairment, iron deposition, oxidative stress, and apoptosis and promoted neuronal regeneration against chronic HAH-mediated neural injury.

**Supplementary Information:**

The online version contains supplementary material available at 10.1007/s11064-022-03611-2.

## Introduction

Human activities in areas of high altitude (more than 3000 m) have recently increased significantly [1]. Among the 140 million people worldwide living permanently at high altitudes [2, 3] and others for tourism or defending boundaries, 5–10% are at risk of developing chronic mountain sickness which is characterized by excessive erythrocytosis and severe hypoxemia [4, 5]. The brain, as one of the most oxygen-consuming organs, is sensitive to hypoxia [6]. Additionally, high-altitude hypoxia (HAH) severely disturbs the structural integrity of the principal neurons and mitochondrial morphology in the hippocampus [7]. The symptoms induced by chronic exposure to an HAH environment include headache, dizziness, sleep disturbances, fatigue and lack of mental concentration [5, 8, 9]. Furthermore, HAH can also trigger neurocognitive dysfunctions such as spatial learning, memory and mood [10]. The treatment of HAH-induced neural injury has thus become a focus of attention in the field of high-altitude medicine [11, 12].

It is of great significance to find suitable formulations for the prevention of HAH-induced brain injury. Green tea leaves contain (−)-epigallocatechin-3-gallate (EGCG) (50–60%), (−)-epigallocatechin (EGC) (15–20%), (−)-epicatechin-3-gallate (ECG) (10–15%) and (−)-epicatechin (EC) (5–10%) [13, 14]. EGCG, which is reportedly to be more abundant in green tea leaves (7.1 g per 100 g) than in oolong tea (3.4 g per 100 g) and black tea leaves (1.1 g per 100 g) [15], has a potent antioxidant property due to the eight hydroxyl groups and two triphenolic groups in its basic structure [16, 17]. Additionally, it has been demonstrated to be able to cross the blood–brain barrier and reach the brain parenchyma in animal studies [18, 19]. There have been some reports on the neuroprotective mechanisms of EGCG such as metal chelation properties, suppression of oxidative stress, inflammation, apoptosis and acceleration of nerve regeneration [20–22]. Zhang et al. reviewed the effect of EGCG on many diseases, and pointed that EGCG protected neuronal cells by inducing autophagy. They also summarized that anti-inflammatory and antioxidant properties of EGCG were vital for its protective role in central nervous system diseases [23].

However, few studies have reported the neuroprotective effect of EGCG against chronic HAH-mediated neural injury. To fill this gap, in this study, we established a rat model of chronic exposure to a natural HAH environment in an attempt to verify the potential treatment mechanism of EGCG. Furthermore, we utilized quantitative susceptibility mapping (QSM), by gradient-echo MRI at 7 T, which can overcome the nonlocal effect of the magnetic field and provide a contrast mechanism for tissues in vivo, to quantify brain iron content [24].

## Materials and Methods

### Animals

A total of 120 male Sprague–Dawley rats weighing 130–150 g was obtained from Chengdu Dashuo Laboratory Animal Co., Ltd. They were kept in an animal house at 18–22 °C in a 12 h light/dark cycle with food and water provided ad libitum. All procedures that performed on animals were approved by the Animal Care and Use Committee of West China Hospital.

### Study Design

Rats were randomized divided into four groups. Rats in the hypoxia group and the h-EGCG group were fed and housed in Yushu, China at an altitude of 4500 m. Rats in the normal altitude group (n group) and the n-EGCG group were fed in Chengdu, China at an altitude of 500 m. Those rats were given a normal feed for one month, followed by different treatments. (1) hypoxia group: rats were intraperitoneally injected every day with physiological saline (0.9%) for one month. (2) h-EGCG group: rats were intraperitoneally injected every day with 50 mg/kg EGCG (purity, 98%; Cas, 989-51-5; Sigma-Aldrich; stored at 4 °C) for one month. (3) n group: rats were intraperitoneally injected every day with physiological saline (0.9%) for one month. (4) n-EGCG group: rats were intraperitoneally injected every day with 50 mg/kg EGCG for one month.

The EGCG (5 mg/mL) was dissolved in water at the ratio of 1:1. The volume that injected to rats was determined by the weight of rats, which reached a final injection amount at 50 mg/kg. After those treatment, some rats were used for Morris water maze assay (n = 10 for each group), brain MRI analysis (n = 10 for each group), respectively. Some other rats were sacrificed and brain tissues were collected for DAB enhanced Perls’ staining (n = 3 for each group), for Western blotting, biochemical assessments and qPCR assays (n = 6 for each group), and transmission electron microscope assay (n = 1 for each group).

### Behavioral Experiment

Morris water maze (MWM) was carried out as previously described to analyze learning and memory of rats [25], it was performed at the same location where the rats were housed. The MWM consisted of a round steel pool (160 cm in diameter, 60 cm in height and 31 cm in depth) filled with water to a level of 1 cm above the top of a platform (10 cm in diameter and 30 cm in depth). The water temperature was maintained at 22 ± 2 °C and opacified with brilliant black ink. The platform was fixed in one of the four quadrants set up during the training for four consecutive days. The trial concluded once the rats reached the platform. The escape latency was recorded. If the rats failed to reach the platform within 60 s, they were then manually guided to the platform and allowed to remain on it for 15 s. On the fifth day, after the platform was removed, the rats entered the quadrant opposite to that of the original platform. Next, the number of platform crossings and movement paths were recorded within 60 s.

### MRI Protocol

QSM was evaluated by MRI, which has been reported in previous study [26].MRI was performed on a 7 Tesla scanner (BioSpec 70/30, Bruker, Germany). A three-dimensional (3D) multiecho gradient-recalled echo (GRE) sequence was utilized for QSM. The experimental parameters were set as follows: repetition time (TR) = 60 ms, flip angle = 15°, slice thickness = 23 mm, acquisition matrix size = 256 × 256, field of view (FOV) = 32 mm × 32 mm, echo time of first echo (TE1) = 5 ms, echo spacing (ΔTE) = 5.77 ms, number of echoes = 8, and bandwidth = 50 kHz. Both magnitude and phase images were saved for QSM reconstruction. There were three steps in the QSM mapping algorithms performed with MATLAB R2014a (The Math Works, Natick, MA), unwrapping the wrapped phase, removing the back ground field and generating susceptibility maps from the tissue field.

### Tissue Preparation

The rats in each group were intraperitoneally administered 10% chloral hydrate for deep anesthesia (1.5 mg/kg) and then transcardially perfused with ice-cold saline (approximately 30 min) by a peristaltic pump (BT100-2 J, LongerPump, Shanghai, China). For DAB enhanced Perls’ staining, the brains were dissected and post-fixed in 2.5% paraformaldehyde overnight at 4 °C. The next day, coronal sections were taken from the hippocampus for staining. For other determinations apart from immunohistochemistry, the hippocampus was stripped rapidly on ice and stored at − 80 °C.

### DAB Enhanced Perls’ Staining

DAB enhanced Perls’ staining was used to detect cellular iron accumulation [27]. Sections of brain tissue were immersed in distilled water for 3 min and then incubated with freshly prepared Perls’ solution (2% potassium ferrocyanide/2% hydrochloric acid) for 30 min, followed by phosphate-buffered saline (PBS) washes. Endogenous peroxidase activity was blocked with 0.3% hydrogen peroxide solution in methanol for 15 min, followed by 3 washes in PBS. Signals were developed by incubation for 3 min in 3,3-diaminobenzidine (DAB) and hematoxylin (Sigma-Aldrich) was used for counterstaining.

### Biochemical Assessments

The hippocampus was isolated and stirred evenly to obtain a 10% homogenate which was then centrifuged at 30,000–40,000 RPM for 10 min to obtain a supernatant to estimate the hippocampal iron by a colorimetric kit (E-BC-K139S, Elabscience, Wuhan, China), and malondialdehyde (MDA) was detected in supernatant by using an ELISA kit (Elabscience, Wuhan, China).

### Western Blotting

The protein concentration was determined using a bicinchoninic acid assay (BCA, Biosharp, Beijing, China) kit. The proteins were separated by 12% SDS-PAGE and then transferred to polyvinylidene fluoride (PVDF) membranes. The latter were blocked for 2 h at room temperature in 5% skimmed milk powder diluted with buffer, and then incubated with primary antibodies overnight at 4 °C, including rabbit anti-cleaved caspase-3 (1:1000, Affinity Biosciences, Jiangsu, China), rabbit anti-brain-derived neurotrophic factor (BDNF) (1:1000, Affinity Biosciences, Jiangsu, China) and rabbit anti-Actin (1:5000, Affinity Biosciences, Jiangsu, China). The next day, the membranes were washed three times with TBST for 5 min each time, and then incubated with an HRP-labeled goat anti-rabbit secondary antibody solution (1:10,000, Servicebio, Wuhan, China) for 1 h, and washed three times for 5 min.

### Quantitative Real‐Time PCR

Animal Total RNA Isolation Kit (Foregene), 5 × All-In-One MasterMix (with AccuRT Genomic DNA Removal kit) (abm) and EvaGreen Express2 × qPCR MasterMix-No Dye (abm) were used in accordance with the instructions of the manufacturers. The specific pairs of primers were as follows: Fpn, forward primer, 5ʹ-CACCACAGGATATGCTTACACTCAGG-3ʹ; reverse primer, 5ʹ-GAGAACAGACCAGTCCGAACAAGG-3ʹ; b-actin, forward primer, 5ʹ-TGTCACCAACTGGGACGATA-3ʹ; reverse primer: 5ʹ-GGGGTGTTGAAGGTCTCAAA-3ʹ. The Fpn mRNA level of each sample was normalized to that of the b-actin mRNA.

### Transmission Electron Microscopy (TEM)

The hippocampi were post-fixed in 2.5% glutaraldehyde electron microscope stationary liquid and then dehydrated in acetone solutions at increasing concentrations and embedded with Epox 812. Then, the sections were stained with uranyl acetate and lead citrate. Ultrastructural images in the CA3 field of the hippocampus were then captured with a transmission electron microscope (TEM) with a JEM-1400-FLASH (JEOL, Tokyo, Japan).

### Statistical Methods

Data are presented as the mean ± standard deviation (SD). Variables that met the parametric test conditions were evaluated by using Welch's t test, one-way or two-way repeated measurement analysis of variance (ANOVA), followed by the Tukey’s multiple comparison test. Those variables that do not met the parametric test conditions were evaluated by using Mann–Whitney U test, Kruskal–Wallis test or Welch and Brown-Forsythe ANOVA. A value of *P* < 0.05 was considered statistically significant. Statistical analysis and figures were obtained by GraphPad Prism Version 9.0 (GraphPad Software, CA, USA).

## Results

### Effect of EGCG on Learning and Memory in Rats Exposed to Chronic HAH

**Fig. 1:**
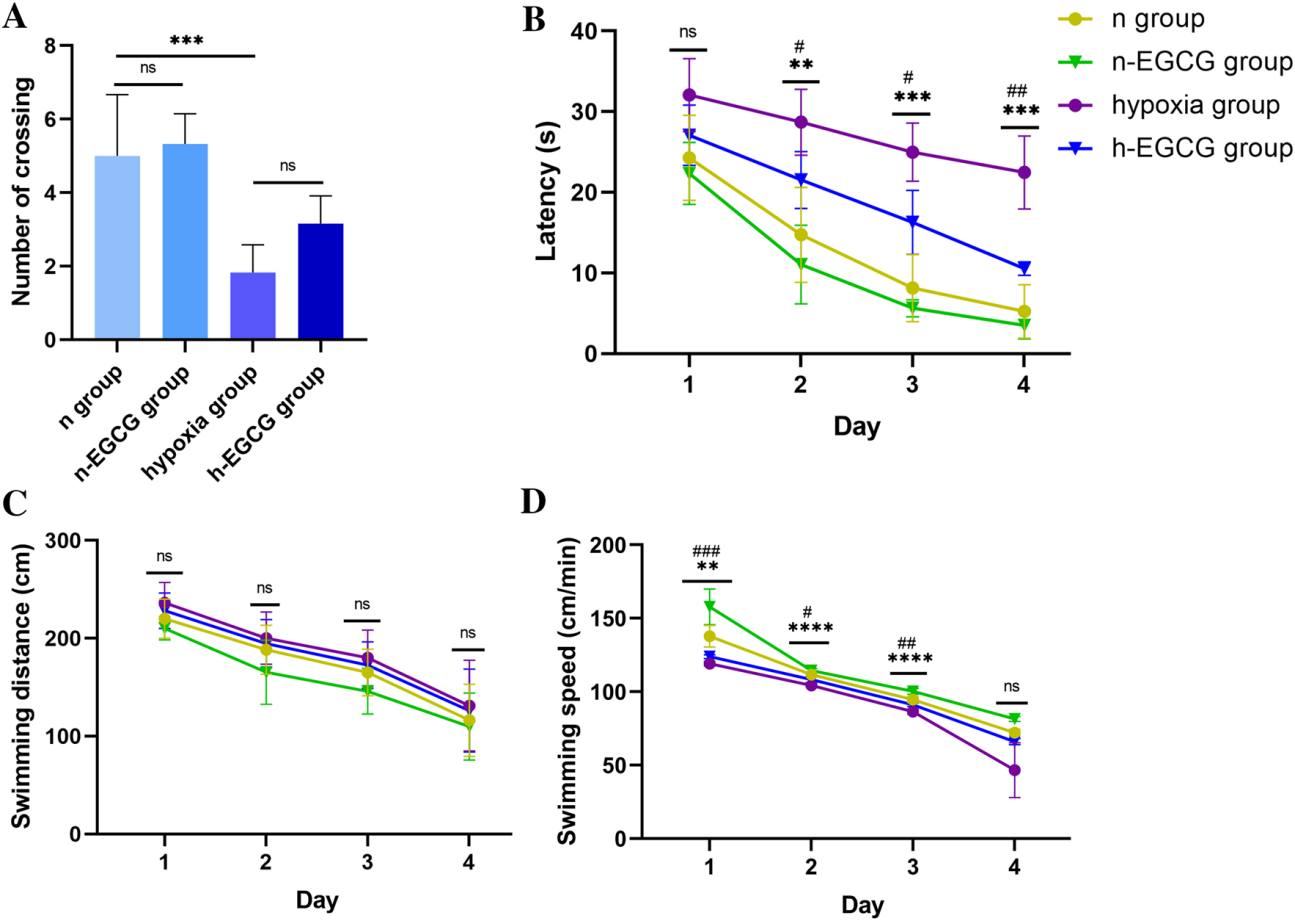
Evaluation of learning and memory performance in rats by the MWM test (n = 10 for each group). **A** Number of platform crossings during a 60 s probe trial of the MWM test. **B** Escape latency in the MWM test plotted against the training days. The results are expressed as the mean ± SD. **C** Swimming distance and **D** Swimming speed. Data are expressed as the mean ± SD. The hypoxia group compared to the n group (**P < 0.01, ***P < 0.001, ****P < 0.0001). The hypoxia group compared to the h-EGCG group (#*P* < 0.05, ##*P* < 0.01). ns, no significance. The difference among number of crossings time in different groups were analyzed by using one-way analysis of variance (ANOVA) and post hoc Tukey’s multiple comparison test. The other differences among these groups were analyzed by using two-way analysis of variance (ANOVA) and post hoc Tukey’s multiple comparison test. EGCG, Epigallocatechin-3-gallate; MWM, Morris water maze

To verify whether EGCG has an effect on the spatial learning and memory performance of rats exposed to chronic HAH, the MWM test was performed. As shown in Fig. 1A, the number of platform crossings by the hypoxia group significantly decreased (*P* < 0.001, vs. the n group). The treatment of EGCG did not affect the number of crossings of rats in both normal altitude group (n group vs. n-EGCG group, *P* > 0.05) or HAH group (hypoxia group vs. h-EGCG group, *P* > 0.05). Next, the escape latency was calculated as the time taken by the rat to reach the hidden platform (Fig. 1B). The results showed that on the second to fourth day of training, the escape latency of rats in the hypoxia group was increased significantly (all *P* values < 0.01, vs. the n group). Moreover, h-EGCG group showed reduced escape latency than that in hypoxia group (all *P* values < 0.05). Also, the swimming speed and distance in MWM were also measured. No significant difference was found among these four group at different time point (All *P* values > 0.05, Fig. 1C). Then, we found that the swimming speed of rats in hypoxia group reduced when compared with n group at second day and third day (all *P* values < 0.01, Fig. 1D). Also, the swimming speed of rats in h-EGCG group were higher than that in hypoxia group (all *P* values < 0.05, Fig. 1D).

### DAB Enhanced Perls’ Staining

**Fig. 2:**
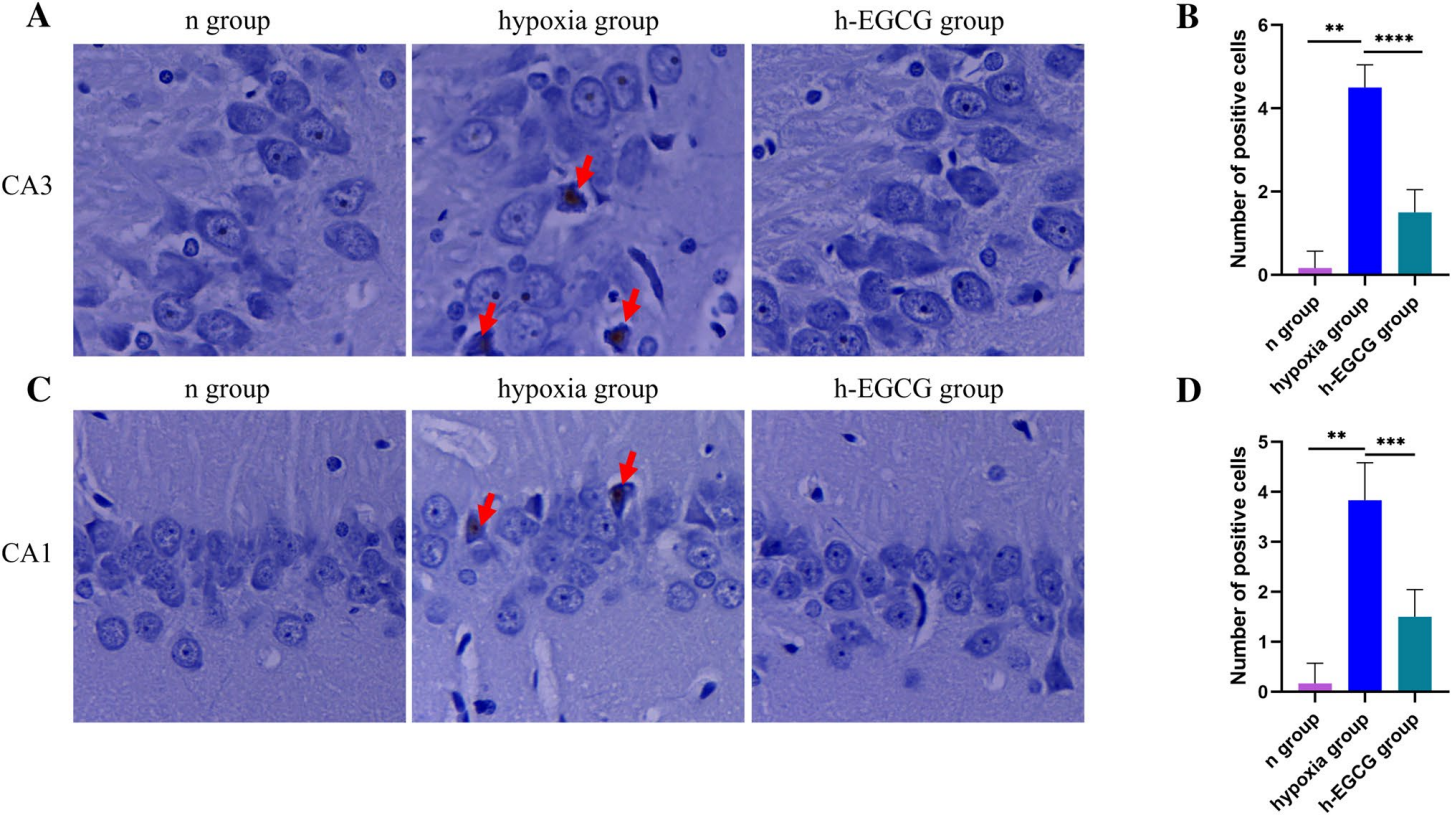
DAB enhanced Perls’ staining of the rats (n = 3 for each group). **A** Field of interest and representative images of Perl's staining in CA3 of the n group, hypoxia group and h-EGCG group. **B** The amount of iron accumulation in CA3. **C** Field of interest and representative images of Perl's staining in the hippocampal CA1 region of the three groups. **D** The amount of iron accumulation in CA1. Red arrow, Perls’ staining positive cells. Data represent the mean ± SD. **P < 0.01, ***P < 0.001, ****P < 0.0001. Mann–Whitney U test was used for analyzing difference between n group and hypoxia group, Welch's t test was used for hypoxia group and h-EGCG group. *DAB* diaminobenzidine

After 8 weeks of chronic HAH exposure, iron increased significantly in CA1 and CA3 areas of the hippocampus of the brain compared with that of the n group (All *P* values < 0.01). After EGCG intervention, iron accumulation in CA3 and CA1 of the hippocampus were reduced compared to that of the hypoxia group (*P* < 0.0001, *P* < 0.001, respectively; Fig. 2).

### Magnetic Susceptibility Changes in Hippocampal Regions

**Fig. 3:**
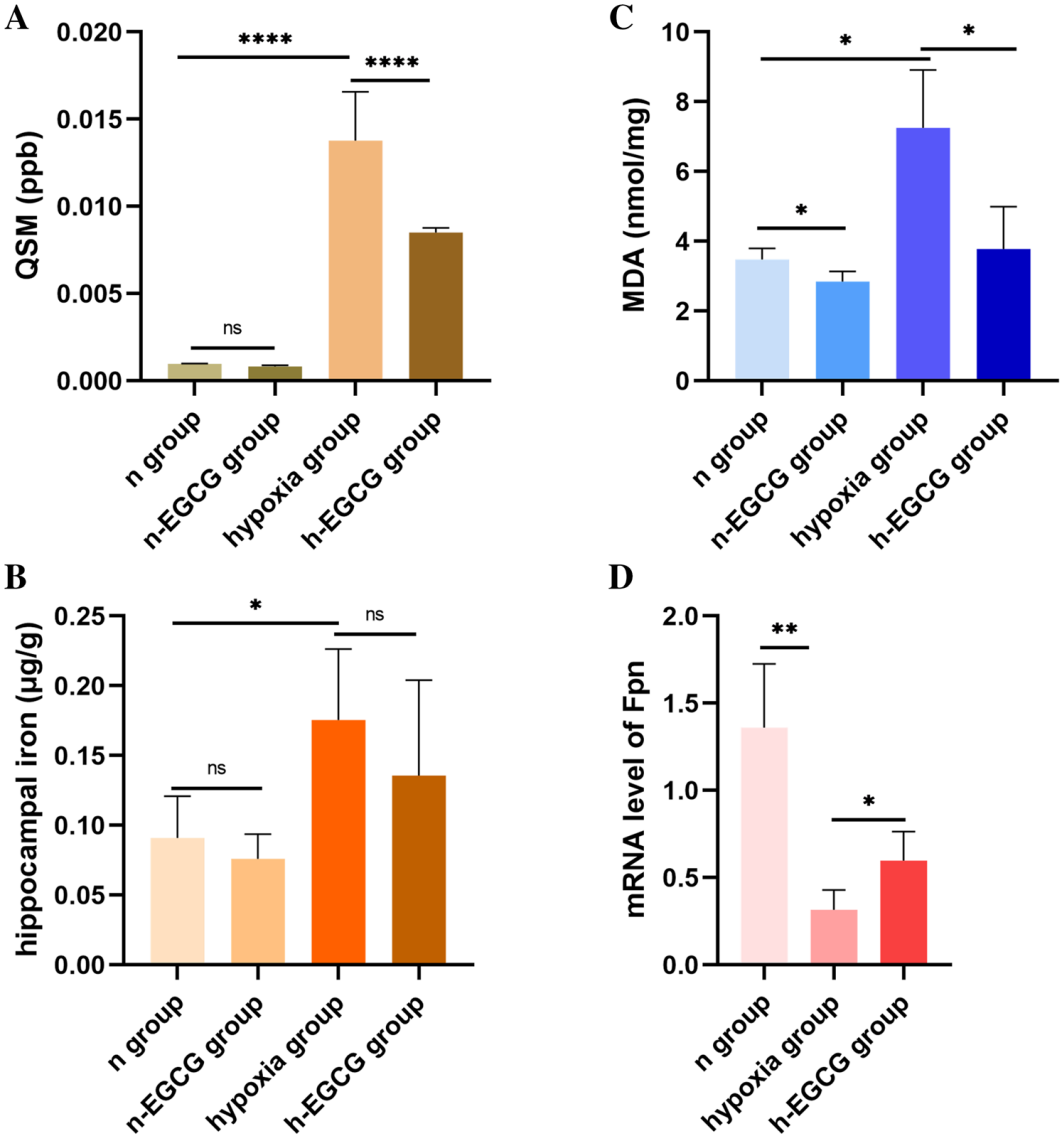
HAH increased the content of iron accumulation and oxidative stress. **A** QSM values evaluated by MRI. QSM, quantitative susceptibility mapping (n = 10 for each group). **B** Iron concentration of supernatant from hippocampal homogenate (n = 6 for each group). **C** Concentration of MDA in supernatant from hippocampal homogenate (n = 6 for each group). **D** mRNA levels of Fpn in hippocampus (n = 6 for each group). Data are expressed as the mean ± SD. *P < 0.05, **P < 0.01, ****P < 0.0001. *ns* no significance. Ordinary one-way ANOVA post hoc Tukey’s multiple comparison test was used to analyze the difference of QSM. Kruskal–Wallis test was used to analyze the difference of iron in hippocampus. Welch and Brown-Forsythe ANOVA was used to analyze the difference of MDA and Fpn. MDA, malondialdehyde

The susceptibility values in the hippocampus increased significantly after HAH exposure in comparison with that of the n group (*P* < 0.0001, Fig. 3A). After EGCG intervention, the values decreased compared to those of the hypoxia group (*P* < 0. 0001, Fig. 3A).

### Effects of EGCG on Hippocampal Oxidative Stress, Iron and Fpn

Compared to that of the n group, the MDA and iron contents of the hypoxia group were elevated (*P* < 0.05, Fig. 3B, C). EGCG treatment reduced the levels of MDA and iron, indicating that alleviation of oxidative stress may facilitate EGCG to play a protective role in chronic HAH-induced brain injury in rats. To understand the mechanisms by which brain iron contents were changed by HAH, we further examined the mRNA expression of hippocampal Fpn and found that the Fpn decreased in the hypoxia group and increased in the h-EGCG group (*P* < 0.01, *P* < 0.05, respectively, Fig. 3D).

### Western Blotting Results

**Fig. 4:**
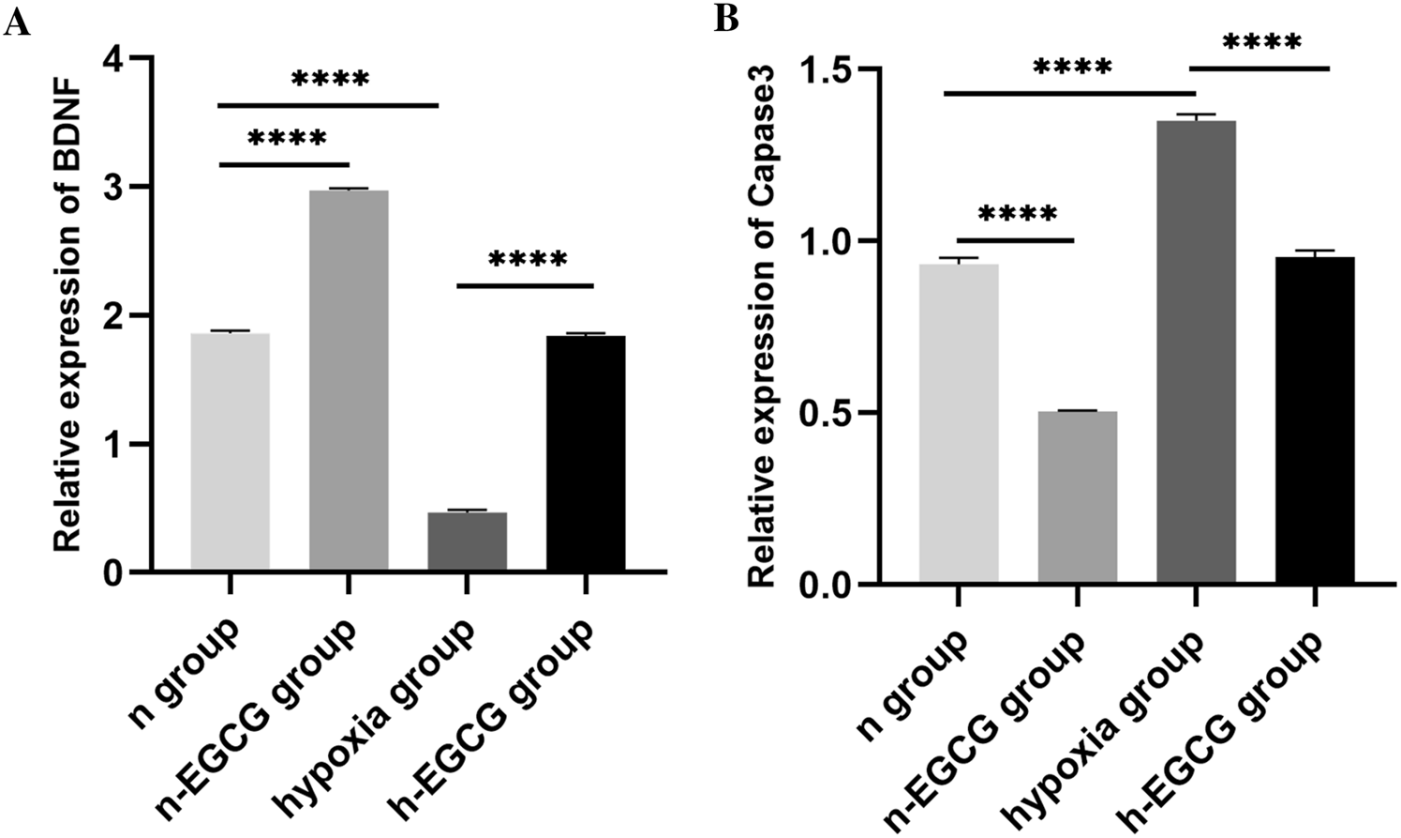
The protein levels of Caspase-3 and BDNF were determined by WB analysis (n = 6 for each group). **A** BDNF protein **B** Caspase-3 protein. Data are expressed as the mean ± SD. ****P < 0.0001. Welch and Brown-Forsythe ANOVA was used for statistically analysis. *WB* western blotting, *BDNF* brain-derived neurotrophic factor, *SD* standard deviation

We assessed the effects of EGCG on the Caspase-3 and BDNF levels. Western blotting (WB) analysis showed higher expression of Caspase-3 and lower expression of BDNF in the hippocampus of the hypoxia group than that in the n group (all *P* values < 0.0001, Fig. 4). Moreover, EGCG treatment decreased the levels of Caspase-3 and increased the levels of BDNF (all *P* values < 0.0001, Fig. 4).

### Neural Ultrastructural Changes

**Fig. 5:**
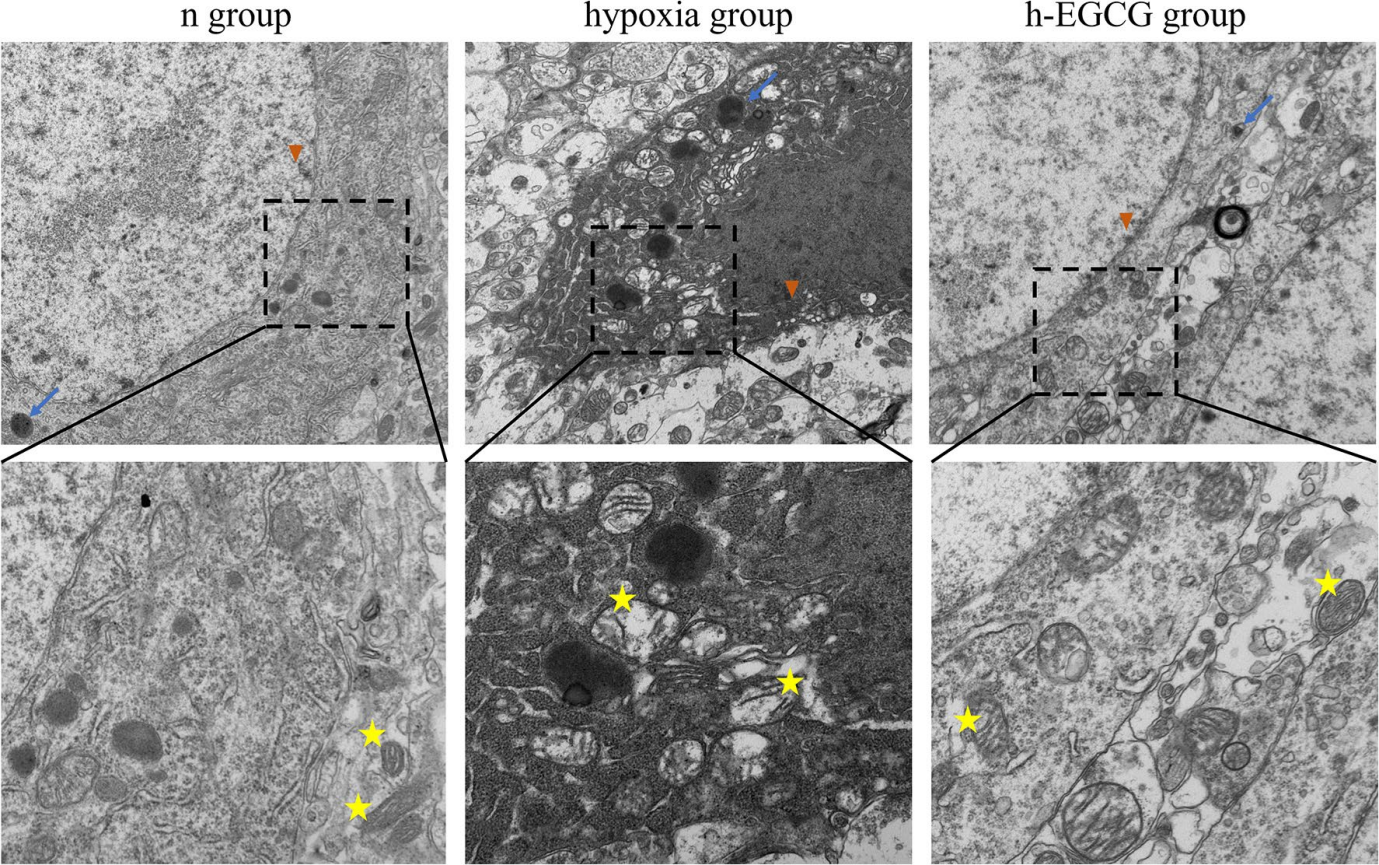
The hippocampal CA3 region of the rats was observed using transmission electron microscope (TEM). The neurons in the hypoxia group presented irregular shapes with nuclear shrinkage, condensed nuclear chromatin, swollen mitochondria and slightly expanded rough endoplasmic reticulum (vs. the n group). In the h-EGCG group, subtle mitochondrial swelling was still found. EGCG, Epigallocatechin-3-gallate. Mitochondria (marked by yellow pentagram), lysosome (marked by blue arrow), condensed nuclear chromatin (marked by red triangle)

After 2 months of chronic HAH exposure, mitochondria in the hippocampal CA3 of the hypoxia group became swollen due to the reduction or breakage of the ridge. Moreover, the hypoxia group rats showed condensed nuclear chromatin, nuclear shrinkage, a high degree of endoplasmic reticulum swelling, increased lysosomes and increased electron density. After treatment with EGCG, mitochondrial swelling, mitochondrial crystal dissolution and electron density were markedly decreased compared to those in the hypoxia group (Fig. 5).

## Discussion

In the present study, we found that treatment with EGCG substantially improved the learning, memory, and spatial exploration abilities of the hypoxia group rats and mitigated the hippocampal neural injury induced by chronic exposure to the HAH environment. Moreover, we also found that, despite significant improvements in all the indicators after EGCG treatment, these indicators seldom returned to normal prehypoxia levels.

Prior studies have found that EGCG has some effects on various types of learning and memory impairment models [28, 29]. Saffar et al. found that EGCG can improve the memory of morphine-treated rats [30]. Consistent with their results, in our study, after daily intervention with 50 mg/kg EGCG in rats chronically exposed to HAH, the learning, memory and spatial exploration abilities of the subjects were improved, suggesting that EGCG might ameliorate learning and memory impairments at high altitudes.

Many studies have shown that overproduction of iron in the brain has a neurotoxic effect as it contributes to oxidative damage [31]. In this study, iron accumulation was detected by MRI, immunohistochemistry staining and biochemical assessments. In addition, FPN, as the only known multitransmembrane iron export protein in mammalian cells, was assessed with PCR. The susceptibility values, positive cells of DAB enhanced Perls’ staining, hippocampal iron contents increased in the hypoxia group and decreased in the h-EGCG group while FPN expression showed the opposite trend. Therefore, we concluded that HAH-induced iron accumulation may occur through the inhibition of iron efflux via a reduction in FPN expression.

MDA is the end product of lipid peroxidation [32, 33], and can be used as an indicator of peroxidation. Prior studies have shown that oxidative stress can increase with increasing altitude [34]. Our study also revealed that MDA concentrations increased significantly at high altitudes. In addition, we analyzed the expression of Caspase-3 and BDNF. Caspase-3 is the most important initiator and performer of terminal cleavage enzymes and apoptosis [35], while BDNF, as a neurotrophin, which can regulate neuron survival and differentiation as well as enhance synaptic transmission [36], promotes regeneration. Lin et al. reported that hypoxia can increase the levels of Caspase-3 and induce apoptosis in hippocampal neurons [37]. Lee et al. reported that after 3 min of global ischemia in the gerbil, EGCG prevented ischemia-induced hippocampal cell death [38]. In our study, we also found that EGCG decreased the levels of the proapoptotic protein, Caspase-3 and increased the expression of BDNF.

Mitochondrial structural damage and dysfunction are important pathological features in the brain under hypoxia [39]. In our study, ultrastructural observation detected the presence of deep staining, mitochondrial swelling and crista disappearance in hippocampal neurons under HAH, whereas hippocampal neurons after EGCG treatment were significantly protected from these injuries such as karyopyknosis. Iwona Zwolak summarized that EGCG may protect against heavy metals toxicity through preserving mitochondrial membrane potential, enhancing mitochondrial antioxidant and respiratory functions [40]. We suspect that these mechanisms may also be present in the protective role of EGCG on brain exposure to HAH envitomrnt. In conclusion, EGCG can reduce iron accumulation, lower the oxidative stress level and apoptosis and promote neuronal regeneration, thus ameliorating rat brain impairment induced by chronic exposure to high altitude hypoxia. Therefore, it has the potential to serve as a novel drug to treat and prevent chronic mountain sickness.

## Supplementary Information

Below is the link to the electronic supplementary material.

Supplementary file1 (TIF 380 kb)

## Data Availability

The datasets generated during and/or analysed during the current study are available from the corresponding author on reasonable request.
