# Supplementary figures and images for: Epigallocatechin-3-Gallate Ameliorated Iron Accumulation and Apoptosis and Promoted Neuronal Regeneration and Memory/Cognitive Functions in the Hippocampus Induced by Exposure to a Chronic High-Altitude Hypoxia Environment
Source: Neurochem Res. 2022 May 13;47(8):2254–62. doi: 10.1007/s11064-022-03611-2 (PMC9352632; doi:10.1007/s11064-022-03611-2)

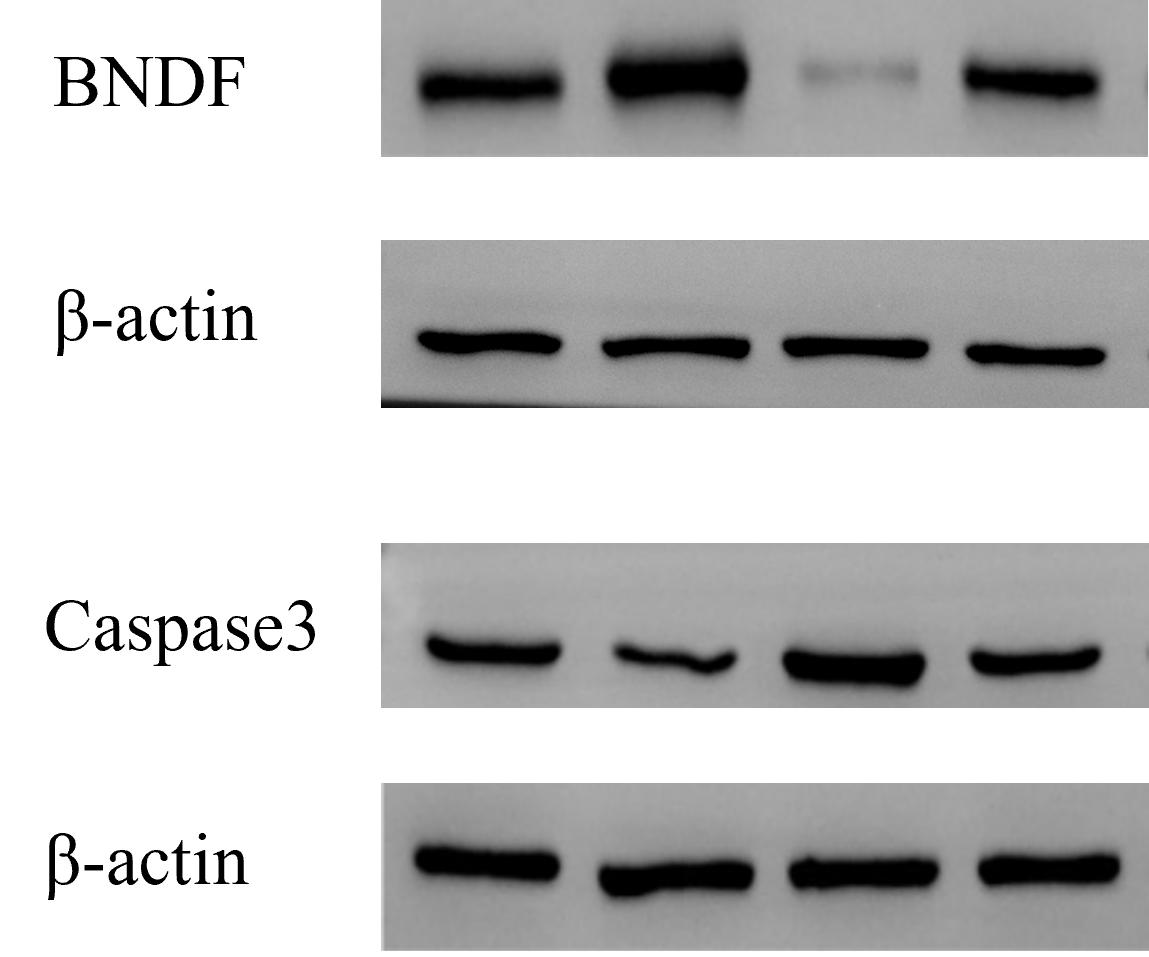

Supplement: Supplementary file 1 — Supplementary file1 (TIF 380 kb) [file 11064_2022_3611_MOESM1_ESM.tif]
